# Systematic Evaluation of a Novel 6-dye Direct and Multiplex PCR-CE-Based InDel Typing System for Forensic Purposes

**DOI:** 10.3389/fgene.2021.744645

**Published:** 2022-01-10

**Authors:** Haoliang Fan, Yitong He, Shuanglin Li, Qiqian Xie, Fenfen Wang, Zhengming Du, Yating Fang, Pingming Qiu, Bofeng Zhu

**Affiliations:** ^1^ Guangzhou Key Laboratory of Forensic Multi-Omics for Precision Identification, School of Forensic Medicine, Southern Medical University, Guangzhou, China; ^2^ School of Basic Medicine and Life Science, Hainan Medical University, Haikou, China; ^3^ First Clinical Medical College, Hainan Medical University, Haikou, China; ^4^ Clinical Research Center of Shaanxi Province for Dental and Maxillofacial Diseases, College of Stomatology, Xi’an Jiaotong University, Xi’an, China; ^5^ Key Laboratory of Shaanxi Province for Craniofacial Precision Medicine Research, College of Stomatology, Xi’an Jiaotong University, Xi’an, China

**Keywords:** InDel, PCR-CE, East Asian population, Hainan Li group, 1000 Genomes Project, Human identification, Intercontinental population differentiation

## Abstract

Insertion/deletion (InDel) polymorphisms, combined desirable characteristics of both short tandem repeats (STRs) and single nucleotide polymorphisms (SNPs), are considerable potential in the fields of forensic practices and population genetics. However, most commercial InDel kits designed based on non-Asians limited extensive forensic applications in East Asian (EAS) populations. Recently, a novel 6-dye direct and multiplex PCR-CE-based typing system was designed on the basis of genome-wide EAS population data, which could amplify 60 molecular genetic markers, consisting of 57 autosomal InDels (A-InDels), 2 Y-chromosomal InDels (Y-InDels), and Amelogenin in a single PCR reaction and detect by capillary electrophoresis, simultaneously. In the present study, the DNA profiles of 279 unrelated individuals from the Hainan Li group were generated by the novel typing system. In addition, we collected two A-InDel sets to evaluate the forensic performances of the novel system in the 1,000 Genomes Project (1KG) populations and Hainan Li group. For the Universal A-InDel set (UAIS, containing 44 A-InDels) the cumulative power of discrimination (CPD) ranged from 1–1.03 × 10^–14^ to 1–1.27 × 10^–18^, and the cumulative power of exclusion (CPE) varied from 0.993634 to 0.999908 in the 1KG populations. For the East Asia-based A-InDel set (EAIS, containing 57 A-InDels) the CPD spanned from 1–1.32 × 10^–23^ to 1–9.42 × 10^–24^, and the CPE ranged from 0.999965 to 0.999997. In the Hainan Li group, the average heterozygote (He) was 0.4666 (0.2366–0.5448), and the polymorphism information content (PIC) spanned from 0.2116 to 0.3750 (mean PIC: 0.3563 ± 0.0291). In total, the CPD and CPE of 57 A-InDels were 1–1.32 × 10^–23^ and 0.999965, respectively. Consequently, the novel 6-dye direct and multiplex PCR-CE-based typing system could be considered as the reliable and robust tool for human identification and intercontinental population differentiation, and supplied additional information for kinship analysis in the 1KG populations and Hainan Li group.

## Introduction

Insertion/deletion (InDel) polymorphisms, the length polymorphisms resulting from the insertion or deletion of one or more nucleotides, are gradually becoming a type of alternative genetic markers for forensic purposes (Bus et al., 2016; Sun et al., 2016; Caputo et al., 2017; Sheng et al., 2018; Xie et al., 2018; Zhang et al., 2018; Sun et al., 2019; Zhang et al., 2019; Abel et al., 2020; Cui et al., 2020; Huang et al., 2020; Zhang et al., 2020). Low mutation rates (∼10^–9^) and no stutter/noise peaks are the overwhelming superiorities for InDels, which possess desirable properties of both short tandem repeats (STRs) and single nucleotide polymorphisms (SNPs) (Chakraborty et al., 1999; Weber et al., 2002; Bhangale et al., 2005; Mills et al., 2006; Pakstis et al., 2007; Mullaney et al., 2010; Pakstis et al., 2010; da Costa Francez et al., 2012; Kidd et al., 2012). In addition, the relatively small amplicon sizes of InDels enhance discrimination efficiencies in some dated or highly degraded samples from crime scenes when compared with STRs (Golenberg et al., 1996; Brinkmann et al., 1998; Jin et al., 2019). With the relatively uncomplicated chemical and operational approaches to detect the length variations in contrast to the determination methods of SNPs (Kwok 2002; Amoako et al., 2017; Matsuda 2017), the detection method by capillary electrophoresis (CE) for InDels could be extensively applied in distinct forensic scenarios (Zhao et al., 2018; Chen et al., 2019; Jin et al., 2019; Tao et al., 2019; Huang et al., 2020; Song et al., 2020; Zhang et al., 2020).

At present, the commercial and widely-used InDel kits present some issues, 1) they are not always suitable for East Asian (EAS) ancestry populations; 2) the insufficient utilization for the CE system; and 3) the time-consuming procedures for DNA extractions and/or purifications. The shortcomings for most InDel typing systems limit the promotion of forensic system effectiveness and the extension of forensic scenarios for EAS populations. Therefore, based on the underlying genome-wide data of the EAS populations from the 1,000 Genome Project (1KG) Phase 3 (Genomes Project et al., 2010; Genomes Project et al., 2015) and the engineering fundamental logic for the maximum utilization of multiplex PCR-CE system, a novel 6-dye direct and multiplex PCR-CE-based typing system with relatively short amplicons (<230 bp), consisting of 57 autosomal InDels (A-InDels), 2 Y-chromosomal InDels (Y-InDels), and Amelogenin, was studied to expand application scenarios for forensic purposes in EAS populations, especially for different Chinese populations. Moreover, forensic efficiencies and population genetic analyses of the direct and multiplex PCR-CE-based InDel typing system were further evaluated in 26 globally dispersed populations and the Hainan Li (HNL) group, which is a relatively isolated Chinese group revealed by the previous studies (Fan et al., 2018b; Fan et al. 2019b; Fan et al. 2021a; Wang et al., 2021).

## Materials and Methods

### Sample and Data Collections

Bloodstain samples of 279 unrelated healthy Hainan Li individuals were collected after receiving their informed consents. The experiment was conducted in accordance with the guidelines of humane and ethical research of Xi’an Jiaotong University and Southern Medical University, and warranted by the Ethics Committee of Xi’an Jiaotong University (No. 2019–1231). To evaluate the universal applicability of the novel 6-dye direct and multiplex PCR-CE-based typing system, we collected the population data of global 1KG populations from five continents.

### Amplification and CE Detection

The amplification of the novel 6-dye direct and multiplex PCR-CE-based typing system was performed in a single multiplex PCR reaction (25 μl in total) using 10 μl of Reaction Mix (AGCU ScienTech Incorporation, Wuxi, Jiangsu, China), 1 μl of U-Taq Enzyme (AGCU ScienTech Incorporation), 5 μl of InDel 60 Primers (AGCU ScienTech Incorporation), and 9 μl of sdH_2_O. PCR cocktail was performed on the GeneAmp PCR System 9700 Thermal Cycler (Thermo Fisher Scientific, Waltham, MA, USA) based on the following parameters: initial denaturation at 95°C for 5 min; then 28 cycles of 94°C for 30 s, 60°C for 1 min, and 62°C for 1 min; and the final extension at 72°C for 10 min. Afterward, 1 μl of PCR production was added to the cocktail of 0.5 μl AGCU Marker SIZ-500 (AGCU ScienTech Incorporation) and 12 μl of HiDi™ formamide (Thermo Fisher Scientific). The mixture was denatured at 95°C for 3 min and then immediately chilled on ice for 3 min. Finally, the product was detected on the 3500xL Genetic Analyzer (Thermo Fisher Scientific) using 36-cm capillary arrays (Thermo Fisher Scientific) with the POP-4® Polymer (Thermo Fisher Scientific). The CE parameters were as follows: 10 s injection at 2 kV, and electrophoresis at 15 kV for 1,400 s at 60°C. Genotyping data for each sample were determined by GeneMapper® ID-X software v1.6 (Thermo Fisher Scientific). Control DNA 9948 and deionized water were used as positive and negative controls, respectively.

### Statistical Analysis

Allele frequencies of InDels were calculated using SAS® 9.4 software (SAS Institute Inc., Cary, NC, USA). The forensic parameters, match probability (MP), power of discrimination (PD), polymorphism information content (PIC), power of exclusion (PE), typical paternity index (TPI), and heterozygote (He), were conducted by PowerStats software (Promega, Madison, WI, USA). The Hardy–Weinberg equilibrium (HWE) and linkage disequilibrium (LD) were evaluated by Arlequin v3.5 (Excoffier and Lischer 2010). The mean values and standard deviations of forensic relevant parameters were calculated by SAS® 9.4 software (SAS Institute Inc., Cary, NC, USA).

Population pairwise genetic distances (i.e., *F*_*ST*_) and corresponding *p-*values between different populations were estimated by analysis of molecular variance (AMOVA) using raw data at Arlequin v3.5 (Excoffier and Lischer 2010). Genetic similarities and differences were further visualized by principal component analysis (PCA) and multidimensional scaling plot (MDS) using *R* (https://www.r-project.org/) based on insertion allelic frequencies. Additionally, phylogenetic relationships among different populations were depicted in Molecular Evolutionary Genetics Analysis 7.0 (MEGA 7.0) software (Kumar et al., 2016) with neighbor-joining method (Saitou and Nei 1987) and visualized by the Interactive Tree of Life v5 (iTOL) (Letunic and Bork 2019). Other high-quality figures all used *R* to visualize.

## Results and Discussion

### Details of the Novel Typing System and Distinct A-InDel Sets

**FIGURE 1:**
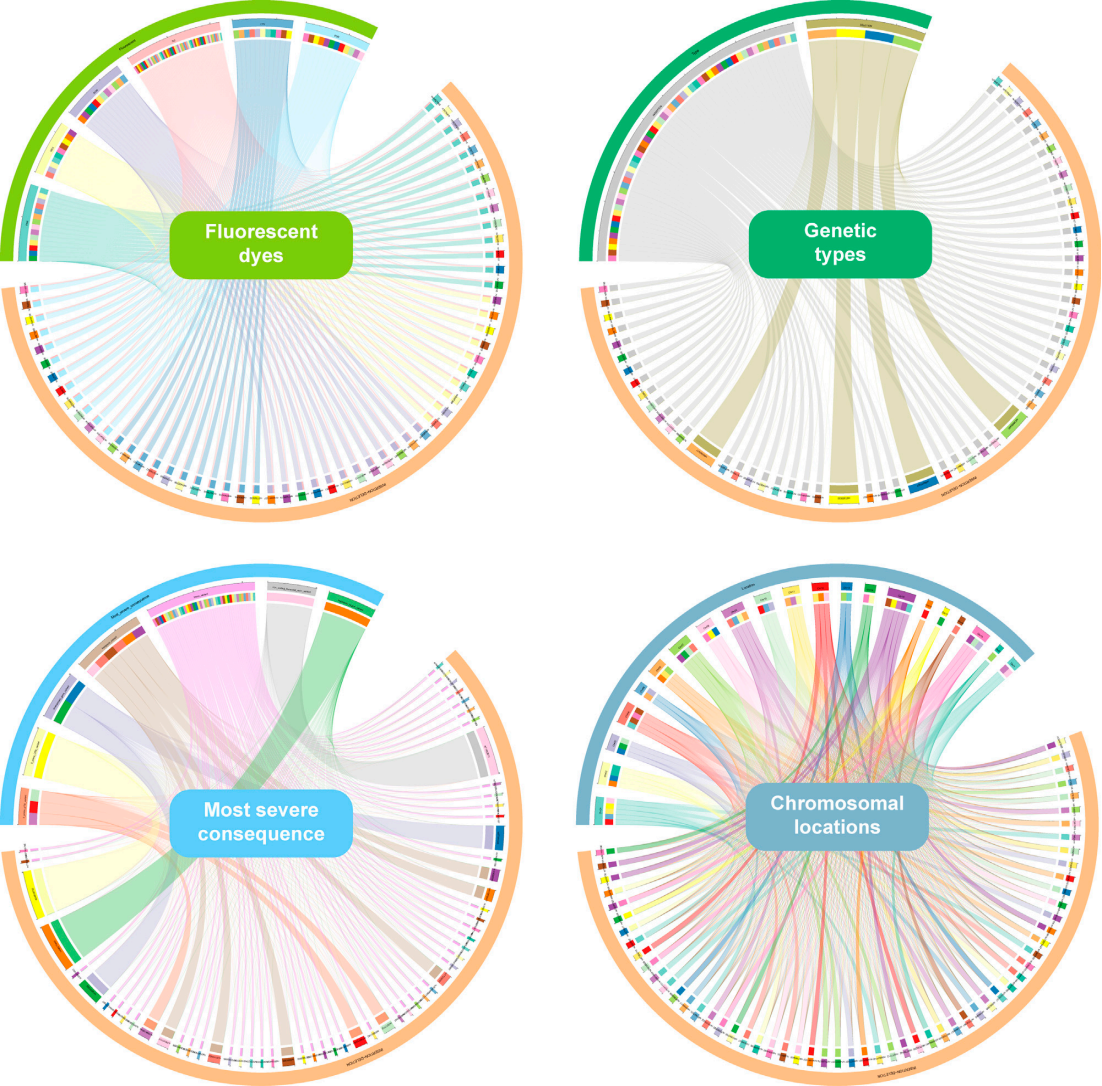
Detailed information of 59 InDels in the direct and multiplex PCR-CE-based typing system.

Supplementary Table S1 presents the detailed InDel information of the 6-dye direct and multiplex PCR-CE-based typing system. All genetic markers are autosomal and Y-chromosomal biallelic variations of InDels with the minimum allele frequency (MAF) ≥0.25 in most EAS populations (CHS and CHB in particular). A total of six fluorescent dyes were applied to the direct typing system. The SIZ dye marked the internal reference item, and the HEX, LYN, SUM, PUR, and FAM dyes labeled 10, 11, 12, 13, and 14 InDels in accordance with the amplicon sizes, respectively (Figure 1 and Supplementary Table S1). Besides, the insertion/deletion fragment lengths ranged from 0 to 12 bp, and the amplicon sizes spanned from 76 to 226 bp, respectively. With the consideration of short amplicons for more forensic application scenarios, further validation and evaluation of forensic efficiencies, such as the ability to analyze dated/degraded samples, sensitivity, specificity, stability, and so on, should be performed in our subsequent study. In addition, the 59 InDels were distributed over 20 autosomes and Y chromosome. Of which, 45 InDels were located at the intron variant regions, 6 InDels were located at the intergenic variant regions, 3 InDels were located at the 3′ UTR variant regions, 2 InDels were located at the downstream gene variant regions, 1 InDel was located at the 5′ UTR variant region, 1 InDel was located at the regulatory region variant region, and 1 InDel was located at the non-coding transcript exon variant region (Figure 1). Physical distances of pairwise InDels located on the same chromosomes indicated that most pairwise InDels on the same chromosomes were more than 10 Mb apart except for the five pairs (rs3067397 and rs10607699, ∼6.3 Mb; rs35464887 and rs76158822, ∼4.1 Mb; rs76158822 and rs5897566, ∼9.3 Mb; rs34419736 and rs77635204, ∼8.8 Mb; and rs72085595 and rs34529638, ∼5.9 Mb), but *p*-values (after Bonferroni corrections) of LD analyses (data not shown) were not statistically significant in the 1KG populations and Hainan Li group.

**FIGURE 2:**
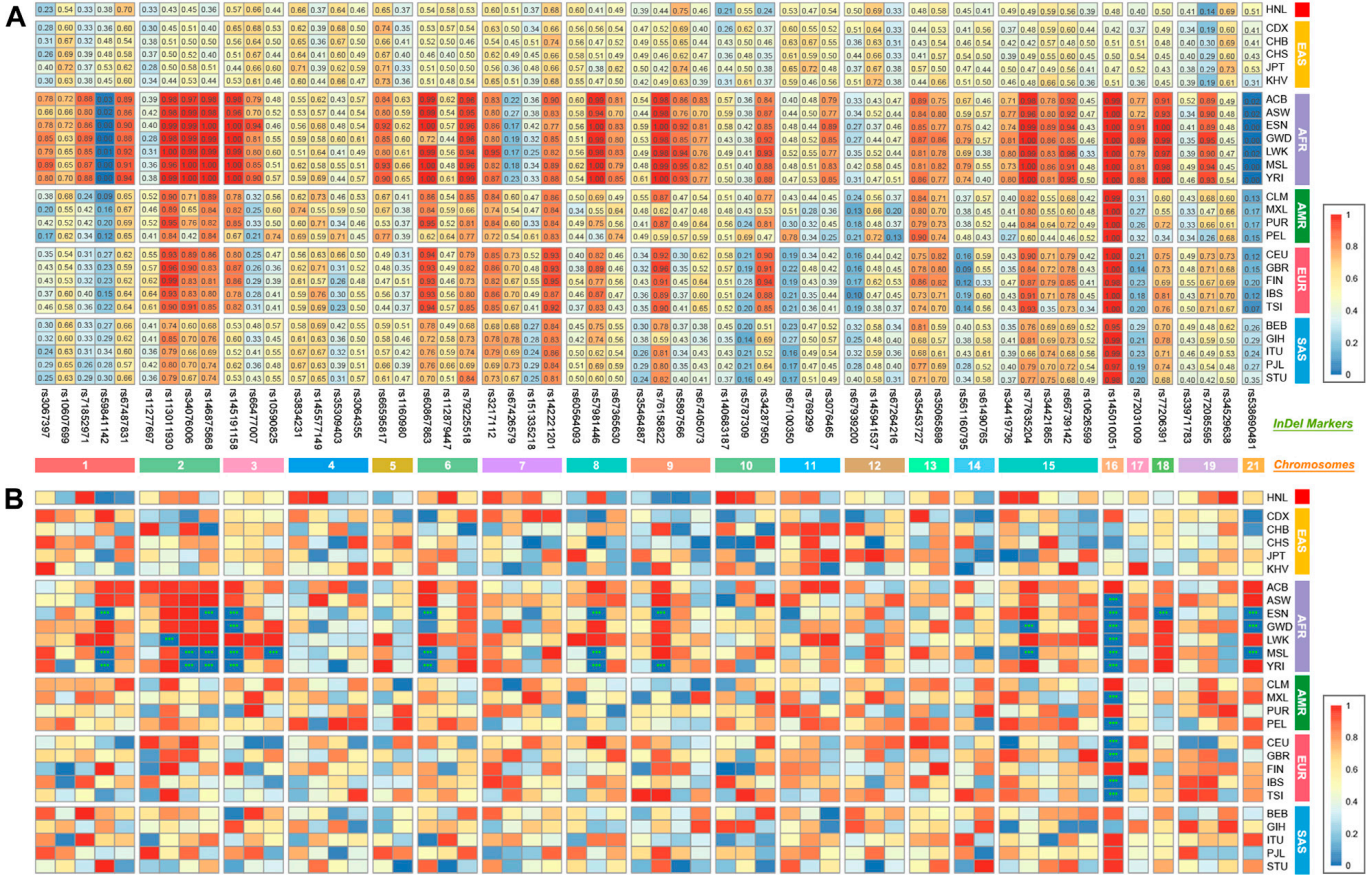
Insertion allelic frequencies and Hardy–Weinberg equilibrium (HWE) tests for EAIS (57 A-InDels) in the 1KG populations and Hainan Li group. **(A)** Heatmap of insertion allelic frequencies; **(B)** results of HWE tests. (***, *p* < 0.05/57 ≈ 0.0009 after Bonferroni correction. EAIS, East Asia-based A-InDel set, including 57 A-InDels.)

The direct and multiplex PCR-CE typing system (57 A-InDels, 2 Y-InDels, and Amelogenin) was studied based on the genome-wide data from the EAS populations. Thus, the East Asia-based A-InDel set (EAIS, including 57 A-InDels) of the typing system would be performing well for forensic purposes in the EAS populations. The results of HWE tests for 57 A-InDels in the 1KG populations and Hainan Li group are presented in Figure 2B and Supplementary Table S3. An overwhelming majority of A-InDel loci conformed to HWE in the 1KG populations and Hainan Li group after Bonferroni correction (0.05/57 = 0.0009), while 13 A-InDels (rs59841142, rs113011930, rs34076006, rs146875868, rs145191158, rs10590825, rs60867863, rs57981446, rs76158822, rs77635204, rs145010051, rs77206391, and rs538690481) failed to pass the HWE tests, which mainly concentrated on African (AFR) ancestry populations. Therefore, we determined Universal A-InDel set (UAIS, including 44 InDels which excluded the 13 A-InDels unconfirmed to HWE) to evaluate the forensic efficiencies for 26 universal 1KG populations and the Hainan Li group.

### Forensic Parameter Evaluations of UAIS (44 A-InDels) in the 1,000 Genomes Project Populations and Hainan Li Group

Allelic frequencies of all 57 A-InDel loci for the 1 KG populations and Hainan Li group are illustrated in Figure 2A and Supplementary Table S4. For UAIS, including a total of 44 A-InDels conformed by Hardy–Weinberg equilibrium and linkage equilibrium, the insertion allelic frequencies ranged from 0.0934 (GBR, rs561160795) to 0.9861 (YRI, rs79225518). The detailed forensic relevant parameters (MP, PD, PIC, PE, TPI, and He) of UAIS in the 1KG populations and Hainan Li group are calculated and summarized in Supplementary Tables S5–S10. In addition, the mean values and standard deviations for all forensic-related parameters in 26 different reference populations from the 1KG and Hainan Li group are shown in Table 1. The MP values (Mean ± Standard Deviation) of UAIS ranged from 0.3931 ± 0.0302 (CHB) to 0.5123 ± 0.1457 (ESN) (mean MP: 0.4386 ± 0.0951). The PIC values spanned from 0.2816 ± 0.0972 (YRI) to 0.3621 ± 0.0140 (CHS) (mean PIC: 0.3297 ± 0.0633). The TPI values varied from 0.8060 ± 0.1767 (YRI) to 1.0049 ± 0.1351 (CHS) (mean TPI: 0.8959 ± 0.1523). The average PE value was 0.1423 ± 0.0643 with a range from 0.1055 ± 0.0733 (YRI) to 0.1883 ± 0.0571 (CHS). For He, the mean value was 0.4243 ± 0.1071 ranging from 0.3486 ± 0.1444 (YRI) to 0.4939 ± 0.0640 (CHS). What is more, the cumulative match probability (CMP), cumulative power of discrimination (CPD), and cumulative power of exclusion (CPE) values of UAIS for the 1KG populations and Hainan Li group are illustrated in Table 2. The CPD values ranged from 1–1.03 × 10^–14^ (LWK) to 1–1.27 × 10^–18^ (CHB), and the CPE varied from 0.993634 (YRI) to 0.999908 (CHS) in the 1KG populations and Hainan Li group.

**TABLE 1:** Comparisons of forensic relevant parameters in East Asia-based autosomal insertion/deletion set (EAIS) and universal autosomal insertion/deletion set (UAIS) for the 1,000 Genome Project (1KG) populations and Hainan Li group.

Population			Match probability (MP)		Power of discrimination (PD)		Polymorphism information content (PIC)		Power of exclusion (PE)		Typical paternity index (TPI)		Heterozygote (He)	
		n	EAIS	UAIS	EAIS	UAIS	EAIS	UAIS	EAIS	UAIS	EAIS	UAIS	EAIS	UAIS
AFR	ACB	96	0.5699 ± 0.2096	0.4805 ± 0.1218	0.4301 ± 0.2096	0.5195 ± 0.1218	0.2478 ± 0.1275	0.3005 ± 0.0792	0.0940 ± 0.0753	0.1166 ± 0.0657	0.7722 ± 0.1898	0.8340 ± 0.1570	0.3098 ± 0.1773	0.3771 ± 0.1262
	ASW	61	0.5472 ± 0.2079	0.4549 ± 0.1049	0.4528 ± 0.2079	0.5451 ± 0.1049	0.2614 ± 0.1270	0.3167 ± 0.0703	0.1032 ± 0.0752	0.1294 ± 0.0624	0.7954 ± 0.1891	0.8657 ± 0.1472	0.3310 ± 0.1736	0.4042 ± 0.1092
	ESN	99	0.6112 ± 0.2368	0.5123 ± 0.1457	0.3888 ± 0.2368	0.4877 ± 0.1457	0.2256 ± 0.1429	0.2831 ± 0.0953	0.0916 ± 0.0849	0.1152 ± 0.0801	0.7616 ± 0.2169	0.8291 ± 0.1933	0.2885 ± 0.1987	0.3627 ± 0.1507
	GWD	113	0.5871 ± 0.2186	0.5005 ± 0.1283	0.4129 ± 0.2186	0.4995 ± 0.1283	0.2373 ± 0.1323	0.2875 ± 0.0855	0.0889 ± 0.0778	0.1093 ± 0.0726	0.7588 ± 0.1966	0.8166 ± 0.1728	0.2953 ± 0.1810	0.3598 ± 0.1339
	LWK	99	0.5935 ± 0.2277	0.4980 ± 0.1417	0.4065 ± 0.2277	0.5020 ± 0.1417	0.2346 ± 0.1378	0.2902 ± 0.0919	0.0926 ± 0.0793	0.1158 ± 0.0717	0.7662 ± 0.2019	0.8314 ± 0.1725	0.2988 ± 0.1891	0.3705 ± 0.1385
	MSL	85	0.6057 ± 0.2293	0.5060 ± 0.1408	0.3943 ± 0.2293	0.4940 ± 0.1408	0.2296 ± 0.1406	0.2859 ± 0.0938	0.0955 ± 0.0954	0.1140 ± 0.0813	0.7738 ± 0.2438	0.8272 ± 0.1943	0.2941 ± 0.1992	0.3620 ± 0.1458
	YRI	108	0.6111 ± 0.2377	0.5123 ± 0.1503	0.3901 ± 0.2386	0.4893 ± 0.1511	0.2246 ± 0.1429	0.2816 ± 0.0972	0.0847 ± 0.0786	0.1055 ± 0.0733	0.7454 ± 0.2014	0.8060 ± 0.1767	0.2787 ± 0.1914	0.3486 ± 0.1444
AMR	CLM	94	0.4545 ± 0.1186	0.4155 ± 0.0614	0.5455 ± 0.1186	0.5845 ± 0.0614	0.3194 ± 0.0765	0.3448 ± 0.0433	0.1340 ± 0.0685	0.1530 ± 0.0586	0.8753 ± 0.1638	0.9218 ± 0.1364	0.4062 ± 0.1238	0.4453 ± 0.0848
	MXL	64	0.4452 ± 0.1045	0.4201 ± 0.0591	0.5548 ± 0.1045	0.5799 ± 0.0591	0.3271 ± 0.0681	0.3450 ± 0.0405	0.1467 ± 0.0693	0.1622 ± 0.0634	0.9062 ± 0.1655	0.9439 ± 0.1493	0.4285 ± 0.1127	0.4570 ± 0.0859
	PEL	85	0.4616 ± 0.1216	0.4207 ± 0.0559	0.5384 ± 0.1216	0.5793 ± 0.0559	0.3151 ± 0.0772	0.3418 ± 0.0403	0.1313 ± 0.0657	0.1528 ± 0.0539	0.8685 ± 0.1580	0.9212 ± 0.1253	0.4022 ± 0.1250	0.4465 ± 0.0805
	PUR	104	0.4491 ± 0.1066	0.4309 ± 0.0705	0.5509 ± 0.1066	0.5691 ± 0.0705	0.3202 ± 0.0681	0.3309 ± 0.0485	0.1286 ± 0.0573	0.1331 ± 0.0524	0.8634 ± 0.1364	0.8756 ± 0.1217	0.4045 ± 0.1063	0.4174 ± 0.0845
EAS	CDX	93	0.3960 ± 0.0351	0.4011 ± 0.0341	0.6040 ± 0.0351	0.5989 ± 0.0341	0.3589 ± 0.0201	0.3559 ± 0.0216	0.1639 ± 0.0558	0.1628 ± 0.0566	0.9480 ± 0.1308	0.9453 ± 0.1323	0.4631 ± 0.0701	0.4611 ± 0.0721
	CHB	103	0.3890 ± 0.0305	0.3931 ± 0.0302	0.6110 ± 0.0305	0.6069 ± 0.0302	0.3641 ± 0.0145	0.3614 ± 0.0154	0.1720 ± 0.0451	0.1703 ± 0.0435	0.9663 ± 0.1059	0.9622 ± 0.1014	0.4769 ± 0.0520	0.4751 ± 0.0508
	CHS	105	0.4022 ± 0.0308	0.4027 ± 0.0322	0.5978 ± 0.0308	0.5973 ± 0.0322	0.3646 ± 0.0132	0.3621 ± 0.0140	0.1959 ± 0.0560	0.1883 ± 0.0571	1.0223 ± 0.1330	1.0049 ± 0.1351	0.5029 ± 0.0622	0.4939 ± 0.0640
	JPT	104	0.3948 ± 0.0289	0.3961 ± 0.0296	0.6052 ± 0.0289	0.6039 ± 0.0296	0.3607 ± 0.0174	0.3575 ± 0.0185	0.1700 ± 0.0507	0.1611 ± 0.0491	0.9618 ± 0.1182	0.9413 ± 0.1143	0.4728 ± 0.0598	0.4618 ± 0.0589
	KHV	99	0.4005 ± 0.0331	0.4050 ± 0.0344	0.5995 ± 0.0331	0.5950 ± 0.0344	0.3612 ± 0.0191	0.3584 ± 0.0202	0.1828 ± 0.0518	0.1805 ± 0.0562	0.9914 ± 0.1225	0.9867 ± 0.1333	0.4884 ± 0.0600	0.4846 ± 0.0650
	HNL	279	0.3986 ± 0.0409	0.4042 ± 0.0440	0.6014 ± 0.0409	0.5958 ± 0.0440	0.3563 ± 0.0291	0.3522 ± 0.0318	0.1639 ± 0.0372	0.1597 ± 0.0399	0.9463 ± 0.0856	0.9366 ± 0.0920	0.4666 ± 0.0560	0.4602 ± 0.0610
EUR	CEU	99	0.4903 ± 0.1374	0.4474 ± 0.0903	0.5097 ± 0.1374	0.5526 ± 0.0903	0.2957 ± 0.0881	0.3241 ± 0.0606	0.1183 ± 0.0722	0.1370 ± 0.0674	0.8375 ± 0.1746	0.8837 ± 0.1599	0.3754 ± 0.1365	0.4147 ± 0.1109
	GBR	91	0.4934 ± 0.1491	0.4454 ± 0.1003	0.5066 ± 0.1491	0.5546 ± 0.1003	0.2922 ± 0.0938	0.3222 ± 0.0674	0.1122 ± 0.0668	0.1309 ± 0.0599	0.8224 ± 0.1625	0.8693 ± 0.1413	0.3656 ± 0.1370	0.4082 ± 0.1045
	FIN	99	0.4897 ± 0.1430	0.4522 ± 0.1064	0.5103 ± 0.1430	0.5478 ± 0.1064	0.2986 ± 0.0920	0.3236 ± 0.0713	0.1256 ± 0.0795	0.1453 ± 0.0754	0.8543 ± 0.1924	0.9027 ± 0.1796	0.3830 ± 0.1460	0.4224 ± 0.1232
	IBS	107	0.4825 ± 0.1317	0.4381 ± 0.0824	0.5175 ± 0.1317	0.5619 ± 0.0824	0.2995 ± 0.0842	0.3288 ± 0.0559	0.1173 ± 0.0667	0.1384 ± 0.0590	0.8353 ± 0.1605	0.8870 ± 0.1384	0.3771 ± 0.1296	0.4212 ± 0.0990
	TSI	107	0.4859 ± 0.1398	0.4356 ± 0.0830	0.5141 ± 0.1398	0.5644 ± 0.0830	0.2971 ± 0.0878	0.3285 ± 0.0551	0.1157 ± 0.0630	0.1333 ± 0.0519	0.8312 ± 0.1530	0.8754 ± 0.1218	0.3748 ± 0.1310	0.4163 ± 0.0914
SAS	BEB	86	0.4245 ± 0.0704	0.4093 ± 0.0444	0.5755 ± 0.0704	0.5907 ± 0.0444	0.3394 ± 0.0472	0.3493 ± 0.0317	0.1520 ± 0.0556	0.1580 ± 0.0497	0.9194 ± 0.1306	0.9335 ± 0.1147	0.4447 ± 0.0837	0.4561 ± 0.0681
	GIH	103	0.4335 ± 0.0917	0.4158 ± 0.0528	0.5665 ± 0.0917	0.5842 ± 0.0528	0.3328 ± 0.0580	0.3449 ± 0.0372	0.1426 ± 0.0590	0.1522 ± 0.0565	0.8967 ± 0.1400	0.9201 ± 0.1313	0.4274 ± 0.1001	0.4451 ± 0.0822
	ITU	102	0.4295 ± 0.0862	0.4169 ± 0.0532	0.5705 ± 0.0862	0.5831 ± 0.0532	0.3345 ± 0.0563	0.3429 ± 0.0392	0.1417 ± 0.0568	0.1487 ± 0.0549	0.8948 ± 0.1337	0.9118 ± 0.1267	0.4274 ± 0.0953	0.4407 ± 0.0796
	PJL	96	0.4305 ± 0.0818	0.4146 ± 0.0513	0.5695 ± 0.0818	0.5854 ± 0.0513	0.3312 ± 0.0526	0.3421 ± 0.0362	0.1330 ± 0.0484	0.1418 ± 0.0463	0.8748 ± 0.1138	0.8959 ± 0.1066	0.4176 ± 0.0855	0.4335 ± 0.0714
	STU	102	0.4250 ± 0.0907	0.4124 ± 0.0626	0.5750 ± 0.0907	0.5876 ± 0.0626	0.3326 ± 0.0563	0.3405 ± 0.0408	0.1242 ± 0.0514	0.1270 ± 0.0531	0.8549 ± 0.1225	0.8621 ± 0.1244	0.4028 ± 0.0893	0.4086 ± 0.0808

**TABLE 2:** Comparisons of forensic system efficiencies in EAIS and UAIS for the 1KG populations and Hainan Li group.

Population			CMP		CPD		CPE	
		n	EAIS	UAIS	EAIS	UAIS	EAIS	UAIS
AFR	ACB	96	3.50E−16	2.82E−15	1–3.50E−16	1–2.82E−15	0.997060	0.996212
	ASW	61	3.28E−17	3.32E−16	1–3.28E−17	1–3.32E−16	0.998356	0.997997
	ESN	99	1.10E−14	3.25E−14	1–1.10E−14	1–3.25E−14	0.996773	0.996199
	GWD	113	1.74E−15	1.56E−14	1–1.74E−15	1–1.56E−14	0.996016	0.994716
	LWK	99	2.44E−15	1.03E−14	1–2.44E−15	1–1.03E−14	0.996858	0.996168
	MSL	85	8.00E−15	2.01E−14	1–8.00E−15	1–2.01E−14	0.997689	0.995993
	YRI	108	8.96E−15	2.55E−14	1–8.96E−15	1–2.55E−14	0.994830	0.993634
AMR	CLM	94	6.44E−21	1.08E−17	1–6.44E−21	1–1.08E−17	0.999771	0.999396
	MXL	64	2.80E−21	1.81E−17	1–2.80E−21	1–1.81E−17	0.999902	0.999636
	PEL	85	1.59E−20	2.00E−17	1–1.59E−20	1–2.00E−17	0.999721	0.999380
	PUR	104	4.54E−21	4.89E−17	1–4.54E−21	1–4.89E−17	0.999654	0.998282
EAS	CDX	93	9.42E−24	2.99E−18	1–9.42E−24	1–2.99E−18	0.999968	0.999637
	CHB	103	3.59E−24	1.27E−18	1–3.59E−24	1–1.27E−18	0.999981	0.999746
	CHS	105	2.40E−23	3.63E−18	1–2.40E−23	1–3.63E−18	0.999997	0.999908
	JPT	104	8.51E−24	1.78E−18	1–8.51E−24	1–1.78E−18	0.999978	0.999593
	KHV	99	1.85E−23	4.59E−18	1–1.85E−23	1–4.59E−18	0.999991	0.999859
	HNL	279	1.32E−23	3.93E−18	1–1.32E−23	1–3.93E−18	0.999965	0.999549
EUR	CEU	99	3.42E−19	1.96E−16	1–3.42E−19	1–1.96E−16	0.999371	0.998665
	GBR	91	3.69E−19	1.38E−16	1–3.69E−19	1–1.38E−16	0.999037	0.998123
	FIN	99	2.90E−19	2.52E−16	1–2.90E−19	1–2.52E−16	0.999627	0.999160
	IBS	107	1.54E−19	8.72E−17	1–1.54E−19	1–8.72E−17	0.999307	0.998716
	TSI	107	1.92E−19	6.68E−17	1–1.92E−19	1–6.68E−17	0.999220	0.998297
SAS	BEB	86	3.34E−22	6.67E−18	1–3.34E−22	1–6.67E−18	0.999927	0.999521
	GIH	103	8.19E−22	1.23E−17	1–8.19E−22	1–1.23E−17	0.999865	0.999368
	ITU	102	5.27E−22	1.39E−17	1–5.27E−22	1–1.39E−17	0.999855	0.999234
	PJL	96	6.30E−22	1.11E−17	1–6.29E−22	1–1.11E−17	0.999731	0.998879
	STU	102	2.46E−22	7.50E−18	1–2.46E−22	1–7.50E−18	0.999529	0.997665

**FIGURE 3:**
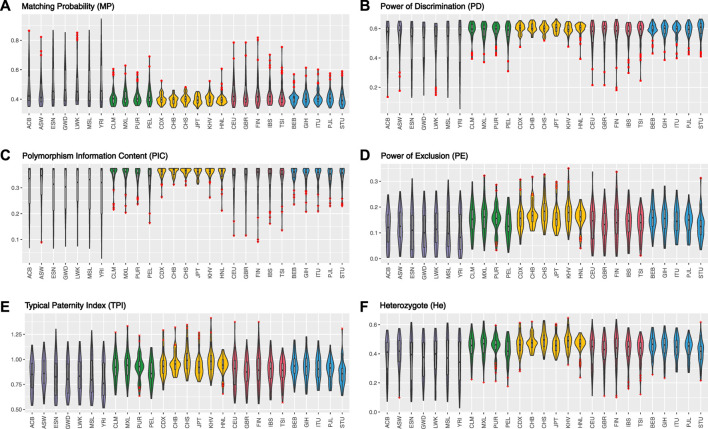
Forensic-related parameters of UAIS (44 A-InDels) for the 1KG populations and Hainan Li group. **(A)** Matching probability; **(B)** power of discrimination; **(C)** polymorphism information content; **(D)** power of exclusion; **(E)** typical paternity index; **(F)** heterozygote.

In total, for UAIS which showed no evidence of deviation from HWE and LDs in both the 1KG populations and Hainan Li group, the forensic-related parameters were distributed relatively balanced (Figure 3), revealing that the UAIS had considerable potential in the field of forensic human identification for universal populations. The 44 A-InDels of UAIS possessed relatively reasonable genetic information (PIC > 0.25) (Botstein et al., 1980), and the UAIS with the CMP range of 1.27 × 10^–18^to 1.03 × 10^–14^ for 27 universal human populations satisfied the requirements for forensic human identification (10^–15^–10^–14^) (Pereira et al., 2009), which indicated that the UAIS could be considered as a powerful tool for human identification. Compared with the CPE provided by the common STR panels (Fan et al., 2019a; Fan et al., 2019b; Li et al., 2020), CPE for UAIS has outclassed 0.993634–0.999908. Therefore, the UAIS could supply additional information for the paternity tests.

### Forensic Parameter Evaluations of EAIS (57 A-InDels) in the East Asian Populations and Hainan Li Group

**FIGURE 4:**
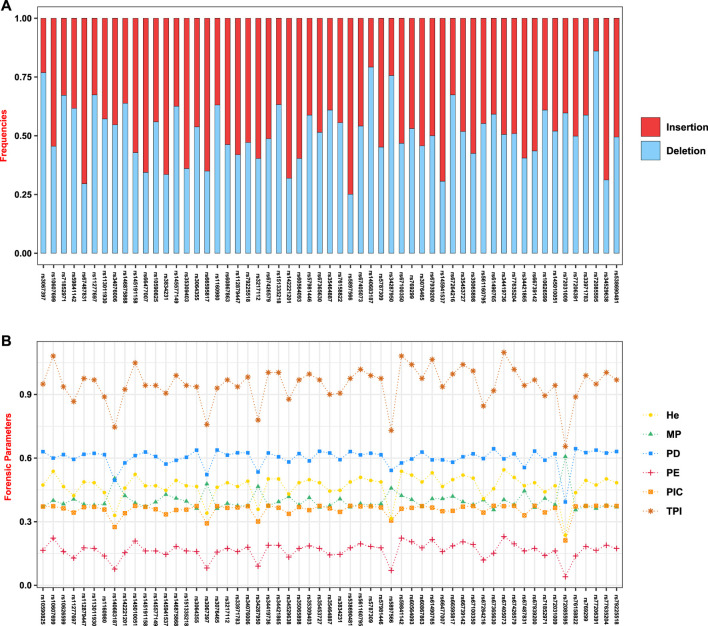
Insertion/deletion allelic frequencies and forensic-associated parameters for EAIS (57 A-InDels) in the Hainan Li group. **(A)** Insertion/deletion allelic frequencies; **(B)** forensic parameters (MP, match probability; PD, power of discrimination; PIC, polymorphism information content; PE, power of exclusion; TPI, typical paternity index; He, heterozygote.

The Hainan Li, inhabiting in the south of Hainan island, is a relatively isolated minority group in China, which is beneficial to clarify the exquisite population structure and develop specific genetic markers for subpopulations in the forensic genetic field (Liu et al., 2020b; Fan et al., 2021a). Hence, a total of 279 healthy Hainan Li individuals were collected for forensic evaluations of 57 A-InDel loci in EAIS. The insertion allelic frequencies of the Hainan Li group are demonstrated in Figures 2A and **4A** and Supplementary Table S4, which were distributed between 0.2079 (rs140683187) and 0.7491 (rs5897566), except for rs72085595 (0.1398). The forensic parameters (MP, PD, PIC, PE, TPI, and He) of the 57 A-InDels in the Hainan Li group are shown in Supplementary Tables S5–S10 and Figure 4B. The MP values of the Hainan Li group ranged from 0.3557 (rs76158822) to 0.6069 (rs72085595) (mean MP: 0.3986 ± 0.0409). The PIC values spanned from 0.2116 (rs72085595) to 0.3750 (rs67939200, rs34419736, rs77206391, and rs538690481) (mean PIC: 0.3563 ± 0.0291). The TPI values varied from 0.6549 (rs72085595) to 1.0984 (rs67405073) (mean TPI: 0.9463 ± 0.0856). The PE values ranged from 0.0405 (rs72085595) to 0.2298 (rs67405073) with an average of 0.1639 ± 0.0372. The He values varied from 0.2366 (rs72085595) to 0.5448 (rs67405073) (mean He: 0.4666 ± 0.0560). Moreover, the CPE and CPD of the 57 A-InDels in the Hainan Li group was 0.999965 and 1–1.32 × 10^–23^, which demonstrated that the EAIS have good performances for individual identification and paternity test in the Hainan Li group (Table 2). What is more, compared with the results of 47 A-InDels in 216 Hainan Li (Liu et al., 2020a) and 30 A-InDels in 207 Hainan Li (Liu et al., 2019), the majority of 57 A-InDels showed more balanced frequency distributions in the same population (Figure 4A). With the number of analyzed A-InDels increased, the CPD and CPE also increased, while the CMP decreased in the Hainan Li group (Table 3).

**TABLE 3:** Comparisons of forensic system efficiencies in different panels with distinct A-InDels for the Hainan Li group (N, number of A-InDel; n, number of population size).

**Population**	**Panel**	**N**	**n**	**CMP**	**CPD**	**CPE**
Hainan Li	Investigator DIPplex kit	30	207	2.92E−11	1–2.92E−11	0.986100
	AGCU InDel 50 kit	47	216	7.67E−18	1–7.67E−18	0.999283
	UAIS	44	279	3.93E−18	1–3.93E−18	0.999549
	EAIS	57	279	1.32E−23	1–1.32E−23	0.999965

**FIGURE 5:**
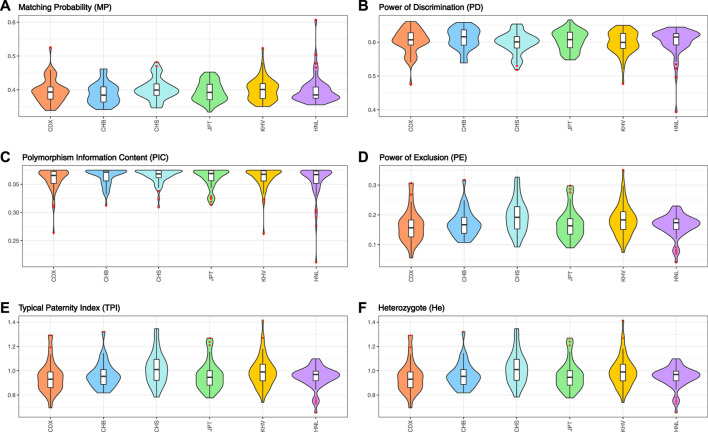
Forensic relevant parameters of EAIS (57 A-InDels) in the East Asian (EAS) populations and Hainan Li group. **(A)** Matching probability; **(B)** power of discrimination; **(C)** polymorphism information content; **(D)** power of exclusion; **(E)** typical paternity index; **(F)** heterozygote.

As shown in Figure 5 and Table 1, the forensic-associated parameters of CDX, CHB, CHS, JPT, and KHV populations, and the Hainan Li group are illustrated. The MP values ranged from 0.3890 ± 0.0305 (CHB) to 0.4022 ± 0.0308 (CHS). The PIC values spanned from 0.3563 ± 0.0291 (HNL) to 0.3646 ± 0.0132 (CHS). The TPI values varied from 0.9463 ± 0.0856 (HNL) to 1.0223 ± 0.1330 (CHS). The PE values ranged from 0.1639 ± 0.0558 (CDX) to 0.1959 ± 0.0560 (CHS). The He values spanned from 0.4631 ± 0.0701 (CDX) to 0.5029 ± 0.0622 (CHS). In addition, the CPE varied from 0.999965 (HNL) to 0.999997 (CHS), and the CPD ranged from 1–1.32 × 10^–23^ (HNL) to 1–9.42 × 10^–24^ (CHB), respectively. The results revealed that the EAIS has sufficient system effectiveness for human identification and kinship analysis in the EAS populations.

### Population Genetic Analyses Among the Hainan Li Group and 1,000 Genomes Project Populations

**FIGURE 6:**
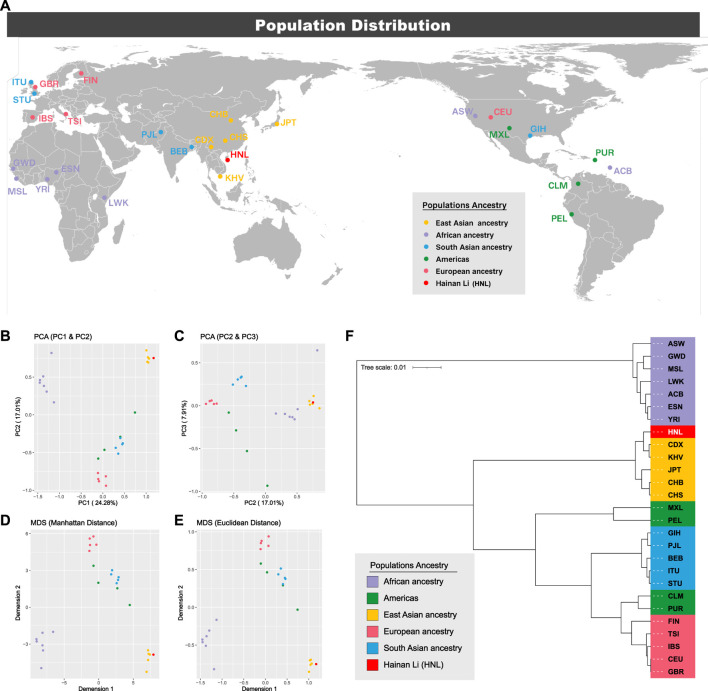
Locations of population distributions and population genetic analyses among the 1KG populations and Hainan Li group. **(A)** Locations of population distributions; **(B)** principal component analysis (PCA) (PC1 and PC2); **(C)** PCA (PC2 and PC3); **(D)** Manhattan-based multidimensional scaling plot (MDS); **(E)** Euclidean-based MDS; **(F)** phylogenetic analysis based on pairwise *F*_*ST*_ values.

To illustrate the genetic landscapes among the 1KG populations and Hainan Li group, the dimensionality reduction analyses (PCA and MDS), which can accelerate the speed of algorithm execution, improve the performance of the analysis model, and reduce the complexity of data at the same time, were conducted based on insertion allelic frequencies of 44 A-InDels, which are illustrated in Figure 2A and Supplementary Table S4. As shown in Figures 6B **, C**, the first, second, and third components (PC1, PC2, and PC3) accounted for 24.28%, 17.01%, and 7.91% of the total variance observed within these populations, respectively. In the PCA diagrams (Figures 6B, C), populations from five different intercontinental ancestries clustered separately, the EAS populations and Hainan Li group clustered together on the upper right. While, the European populations located at the bottom, and the AFR populations distributed on the upper left. In addition, in order to make further confirmation about the genetic relationships between the Hainan Li group and populations from the 1KG conducted by PCA, the Manhattan and Euclidean distance-based MDS were conducted (Figures 5D, E), which also depicted the genetic relationships among the Hainan Li group and 1KG populations. The MDS results (Figures 5D, E) were in accordance with the genetic patterns of PCAs (Figures 5B, C). In brief, the dimensionality reduction analyses (PCA and MDS) made relatively clear distinctions, and the Hainan Li group had the close relationships with the EAS populations.

The results of pairwise *F*_*ST*_ and the corresponding *p*-values between the Hainan Li group and 26 worldwide populations from different continents are listed in Supplementary Table S11. The extreme values of *F*_*ST*_ were identified at CEU and GBR (*F*_*ST*_ = 0.0001, *p* < 0.0001), and GWD and PEL (*F*_*ST*_ = 0.2408, *p* < 0.0001). Phylogenetic relationships between the Hainan Li group and the other 26 reference populations are visualized in the neighbor-joining tree (Figure 6F). The EAS populations and Hainan Li group clustered together. For details, the Hainan Li got together with CDX (*F*_*ST*_ = 0.0037, *p* < 0.0001) and KHV (*F*_*ST*_ = 0.0048, *p* < 0.0001), and CHB and CHS clustered together with JPT in another inner branch. They all belong to Southeast Asia from the perspective of geography. The pairwise genetic distances indicated by *F*_*ST*_ values and the phylogenic relationships based on neighbor-joining tree were consistent with the results of the abovementioned population genetic analyses (PCA and MDS), which manifested that the genetic distances of different populations were consistent with geographic scales in the present study to some degree.

In general, from the perspective of population genetic analyses, compared with paternal Y-STR genetic markers (Fan et al., 2018a; Fan et al., 2018c; Liu et al., 2020b; Ding et al., 2020; Fan et al., 2021b; Fan et al., 2021c; Luo et al., 2021), the novel 6-dye direct and multiplex PCR-CE-based typing system also possessed the ability to differentiate intercontinental populations to a certain extent. The UAIS enabled to make the relatively clear distinctions among populations from five intercontinental ancestries, and the Hainan Li group had the close genetic relationships with EAS populations.

## Conclusion

In conclusion, the direct and multiplex PCR-CE-based typing system was studied based on genome-wide EAS population data, consisting of 57 A-InDels, 2 Y- InDels, and Amelogenin. We collected two A-InDel sets (EAIS and UAIS) according to the numbers of A-InDels, which confirmed to HWE and evaluated the forensic system effectiveness for each set from the perspectives of EAS and global 1KG populations, respectively. For UAIS (44 A-InDels), the CPD ranged from 1–1.03 × 10^–14^ to 1–1.27 × 10^–18^, and the CPE varied from 0.993634 to 0.999908. For EAIS (57 A-InDels), the ranges of CPD and CPE values were 1–1.32 × 10^–23^ to 1–9.42 × 10^–24^, and 0.999965–0.999997, respectively. In addition, the CPD and CPE values of EAIS for the Hainan Li group were 1–1.32 × 10^–23^ and 0.999965, respectively. The population genetic analyses clarified the distinctions among the 1KG populations, and the Hainan Li group had close relationships with EAS populations. Consequently, the novel 6-dye direct and multiplex PCR-CE-based typing system should be considered as a reliable and robust tool for human identification and intercontinental population genetics, and supply additional information for kinship analysis in the 1KG populations and Hainan Li group.

## Data Availability

The data that support the findings of this study are available from the corresponding author upon reasonable request.

## Glossary

InDel: insertion/deletion
STRs: short tandem repeats
SNPs: single nucleotide polymorphisms
1KG: 1,000 Genomes Project
AFR: African
AMR: Americas
EAS: East Asian
EUR: European
SAS: South Asian
HNL: Hainan Li
ESN: Esan in Nigeria
GWD: Gambian in Western Division, Mandinka
**LWK**,: Luhya in Webuye, Kenya
MSL: Mende in Sierra Leone
YRI: Yoruba in Ibadan, Nigeria
ACB: African Caribbean in Barbados
ASW: people with African Ancestry in Southwest United States
CLM: Colombians in Medellin, Colombia
MXL: people with Mexican Ancestry in Los Angeles, CA, United States
PEL: Peruvians in Lima, Peru
PUR: Puerto Ricans in Puerto Rico
CDX: Chinese Dai in Xishuangbanna
CHB: Han Chinese in Beijing
CHS: Southern Han Chinese
JPT: Japanese in Tokyo
KHV: Kinh in Ho Chi Minh City, Vietnam
CEU: Utah residents (CEPH) with Northern and Western European ancestry
GBR: British in England and Scotland
FIN: Finnish in Finland
IBS: Iberian Populations in Spain
TSI: Toscani in Italia
BEB: Bengali in Bangladesh
GIH: Gujarati Indians in Houston, TX, United States
ITU: Indian Telugu in the United Kingdom
PJL: Punjabi in Lahore, Pakistan
STU: Sri Lankan Tamil in the United Kingdom
AMOVA: analysis of molecular variance
***F***_***ST***_: fixation index
MAF: minimum allele frequency
MP: match probability
PD: power of discrimination
PIC: polymorphism information content
PE: power of exclusion
TPI: typical paternity index
He: heterozygote
CMP: cumulative match probability
CPD: cumulative power of discrimination
CPE: cumulative power of exclusion
HWE: Hardy–Weinberg equilibrium
LD: linkage disequilibrium
EAIS: East Asia-based A-InDel Set (57 A-InDels)
UAIS: Universal A-InDel Set (44 A-InDels)
PCA: principal component analysis
MDS: multidimensional scaling plot
CE: capillary electrophoresis
MEGA: Molecular Evolutionary Genetics Analysis
iTOL: Interactive Tree of Life
**SAS** ^®^: Statistical Analysis System
*R*: *R* project for statistical computing
